# A comparison of probe-level and probeset models for small-sample gene expression data

**DOI:** 10.1186/1471-2105-11-281

**Published:** 2010-05-26

**Authors:** John R Stevens, Jason L Bell, Kenneth I Aston, Kenneth L White

**Affiliations:** 1 Department of Mathematics and Statistics, Utah State University, Logan UT 84322, USA; 2 National Agricultural Statistics Service, Columbia MO 65203, USA; 3 Andrology and IVF Laboratories, University of Utah School of Medicine, Salt Lake City, UT 84108, USA; 4 Department of Animal, Dairy, and Veterinary Sciences, Utah State University, Logan UT 84322, USA; 5 Center for Integrated BioSystems, Utah State University, Logan UT 84322, USA

## Abstract

**Background:**

Statistical methods to tentatively identify differentially expressed genes in microarray studies typically assume larger sample sizes than are practical or even possible in some settings.

**Results:**

The performance of several probe-level and probeset models was assessed graphically and numerically using three spike-in datasets. Based on the Affymetrix GeneChip, a novel nested factorial model was developed and found to perform competitively on small-sample spike-in experiments.

**Conclusions:**

Statistical methods with test statistics related to the estimated log fold change tend to be more consistent in their performance on small-sample gene expression data. For such small-sample experiments, the nested factorial model can be a useful statistical tool. This method is implemented in freely-available R code (affyNFM), available with a tutorial document at http://www.stat.usu.edu/~jrstevens.

## Background

### Introduction

Over the past decade, gene expression microarray technology [1] has become a relatively common tool in the biological sciences. A traditional application of this technology is to measure gene expression in two or more groups of individuals that differ according to some characteristic of interest, and then to identify genes whose expression levels change systematically between groups.

An ongoing collaboration with the J. R. Simplot Company involves the cloning of cattle using nuclear transfer (NT). Published results include an evaluation of the differences in NT success rates using cow vs. heifer oocytes [2] and an evaluation of the effects of various time intervals between fusion and activation on the development of bovine NT pregnancies [3]. Recently, microarray technology has been employed in this collaboration, in particular examining the gene expression differences between NT and control (non-NT) samples at various stages of the NT cloning process [4]. One goal of the microarray application in this work is to identify a potential genetic basis for successful NT pregnancies, as well as a potential genetic basis for NT failure. Once understood, this genetic basis can be used to achieve a greater success rate in NT pregnancies, so that attributes such as carcass quality can be maximized and preserved in successive generations of cattle.

Because of the high cost in time and money for NT and gene expression studies, the high level of work necessary to perform NT, and the difficulty in achieving successful bovine NT pregnancies, the sample sizes in these microarray experiments tend to be quite low. While many biomedical applications currently involve dozens of samples, the microarray work done thus far with bovine NT studies has been limited to six to eight samples for each comparison. Much of the statistical methodology that has been developed for microarray data analysis over the past decade [5] has been driven by medical applications such as human cancer research, where samples are much more abundant. As a result, the most common statistical models assume a larger sample size than has been practical in the bovine NT work.

This study evaluates alternative statistical models in an effort to identify appropriate approaches for microarray experiments with small sample sizes. The Results and Discussion section consists of a summary of these methods, including a novel presentation of our probe-level nested factorial model, and a discussion of the relative performance of these methods on spike-in data.

### Data

The relative performance of competing statistical methods can be assessed using spike-in data, and the most promising method was applied to the bovine NT data. For this study, all data considered are of a simple treatment vs. control design.

The motivating bovine NT dataset, mentioned in the Introduction subsection, was taken from an experiment in the cloning of cattle (NT). Tissue samples were taken and prepared from cotyledon (part of the uterine side of the placenta) in an impregnated cow. Four of these pregnancies involved NT and three were control, making 7 total arrays. For these data, we are interested in identifying genes that behave differently in the NT samples than in the non-NT samples. The Affymetrix bovine array was used, with 24,128 probesets.

In spike-in microarray experiments, the "truth" is known a priori. In particular, genes whose mRNA has been artificially increased in a sample to be hybridized onto the array are said to be "spiked-in." The "expression levels" of these genes can be controlled by modifying their spike-in concentrations. Thus their "expression levels" can be made to change across "treatments." That is, they are known to be differentially expressed. Hence it can be verified how well statistical models identify differentially expressed genes in general by how well they find spiked-in genes in these types of experiments.

Data from three spike-in experiments were used to compare the different statistical models. The Affymetrix HGU95A spike-in data [6] consist of 59 arrays and 12,626 probesets on each array, out of which 14 probesets were spiked-in. For this small-sample study, only 8 arrays were used, corresponding to groups M-T of wafer 1532 of the spike-in data, with 4 control and 4 treatment arrays.

The Affymetrix HGU133A spike-in data [6] consist of 42 arrays and 22,300 probesets on each array, out of which 42 probesets were originally spiked-in, with 3 more probesets known to contain probes exactly matching the spiked sequences. Subsequent work [7] identified 22 additional spike-in probesets, but the inclusion of these additional 22 did not substantially change the results of our study. We considered the HGU133A experiment as having 45 spiked-in probesets. For this small-sample study, only 6 arrays were used, corresponding to experiments 5 and 6 of the spike-in data, with 3 control and 3 treatment arrays.

The third and final spike-in dataset used was the "Golden Spike" dataset [8]. There has been some question regarding the credibility of this experiment [9,10]. Specifically, the dataset uses potentially unrealistically high levels of spike-in concentration, and the spike-ins are all up-regulated in the treatment group relative to the control group. However, it is still sometimes used as a tool in comparing statistical models, and we include it here for discussion. This experiment consists of 6 arrays (3 control, 3 treatment), with 1,331 of 14,010 probesets spiked-in.

## Results and Discussion

### Algorithm

The Affymetrix GeneChip is among the most commonly used microarray platforms, and typically represents each gene on the array as a set of 11-20 probes, called a probeset. Preprocessing methods such as RMA [11] combine probe-level intensities to give an expression estimate of each gene in each sample of the experiment. Our initial hypothesis in this study was that using only preprocessed data in assessing differential expression does not take full advantage of available information at the probe level and could be improved. This loss of information seems especially concerning in cases such as the motivating bovine NT study, where there are a relatively small number of samples. Probe-level models for differential expression provide a possible alternative, without this loss of information.

The purpose of this study was to compare several probe-level models with one of the most popular probeset models. In this section, we summarize the approaches of several methods (both probeset and probe-level) to calculate test statistics for evaluating differential expression. Unless otherwise stated, RMA preprocessing [11] was done prior to all probeset models, and prior to all probe-level models, the background correction and quantile normalization steps of RMA were performed. (As an alternative, GCRMA preprocessing [12] was done prior to all probeset models, and prior to all probe-level models, GCRMA background correction [12] followed by quantile normalization was performed.) All analyses were conducted in R [13], making use of several packages in Bioconductor [14].

#### NFM

A nested factorial model (NFM) is a basic structure in experimental design and is developed and presented for the first time in this work with application to microarray data. For each gene *k *we have the model:(1)

*Y*_*ijl *_is the background-corrected quantile-normalized log-scale perfect match intensity for probe *l *of the gene on array *j *under treatment level *i*, *T*_*i *_is the treatment effect, *S*_*j*(*i*) _is the subject (or array) effect nested within treatment, *P*_*l *_is the probe effect, (*TP*)_*il *_is an interaction between treatment and probe, and (*SP*)_*jl*(*i*) _is used as the error term. This model clearly takes into account any probe information that may be lost in a probeset model.

Since we are interested in determining whether or not each gene is differentially expressed between treatment levels, we are testing *H*_0_: *T*_1 _= *T*_2_, and we can look at an F-statistic for the treatment effect. This statistic can be obtained via restricted maximum likelihood (REML) estimation, as implemented in the lme function of the nlme R package [15,16].

It is important to note that, due to the nature of the nested design, an iterative process (REML) is necessary to obtain the F-statistic for each gene. This iterative process takes some time and can be very slow when there is a very large number of factor levels, such as a large number of probes per gene on some array types (as in tiling arrays).

#### PLM

Bolstad (2004) suggested a probe-level model [17], referred to here as PLM. To assess differential expression across treatments, the per-gene model:(2)

is used, where *Y*_*lj *_is the background-corrected quantile-normalized log-scale perfect match intensity for probe *l *of the gene on array *j*, *α*_*l *_is the probe effect, and *β*_*j *_is the array effect. This model is fit using the fitPLM function of the affyPLM R package [17]. Bolstad (2004) defines two test statistics to evaluate differential expression:(3)

and:(4)

Here,  is a vector of estimated array effects and Σ is the portion of the estimated variance/covariance matrix corresponding to . The length of vector *c *is the total number of arrays in the dataset. The elements of *c *depend on how many arrays are in each treatment level. For a simple two-group design, element *j *of *c *is  if array *j *is in treatment group 1,  if array *j *is in treatment group 2, and 0 otherwise, where *n*_*i *_is the number of samples in treatment group *i*. Then *c'* is the log fold change across treatments. PLM is a very fast process since it involves nothing more than some matrix computations, which are very efficient in R.

#### LE

Limma/eBayes (LE) is a very popular probeset-level method in which a hierarchical Bayes model is used to define a moderated test statistic for assessing differential expression [18]. First, we assume a linear model for each gene *k*:(8)

Here, *Y*_*ijk *_is the log-scale expression level for gene *k *on array *j *in treatment group *i*, and *β*_*k*,1 _is the treatment effect. *T*_*i *_is the treatment level of the array, coded as a dummy variable (here, 0 for control and 1 for treatment). The *ε*_*ijk *_is an error term with *Var*(*ε*_*ijk*_) = .

Estimates ,  and *Var*() = *V_k _*are obtained via traditional least squares principles. The traditional t-test statistic for assessing the treatment effect (*H*_0_: *β*_*k*,1 _= 0) is:(12)

Here,  is the element of *V*_*k *_corresponding to the variance of *β*_*k*,1_. Under this *H*_0_, *t*_*k *_follows a t-distribution with degrees of freedom *d*_*k*_.

One limitation of this traditional t-test statistic is the reliability of the estimate . It is intended to estimate the amount of variability expected in the gene's expression levels for all possible samples in a single group (treatment, for example). The small sample sizes sometimes seen in microarray experiments affect the reliability of this estimate, and several statistical models have proposed modified estimates, some of which seek to pool information across genes. The limma/eBayes pooling approach is to assume a prior distribution on the precision:(14)

where  and *d*_0 _are estimated from the data (using all genes) with empirical Bayes methods. The posterior mean:(16)

is used as an updated estimate of the variability . With this, a moderated t-statistic for each gene *k *is:(17)

Under the *H*_0_,  follows a t-distribution with degrees of freedom *d*_0 _+ *d*_*k *_. LE is a very efficient method in the sense that it runs as fast as PLM. The LE method is implemented in the R package limma [18].

#### PLLM

**Figure 1:**
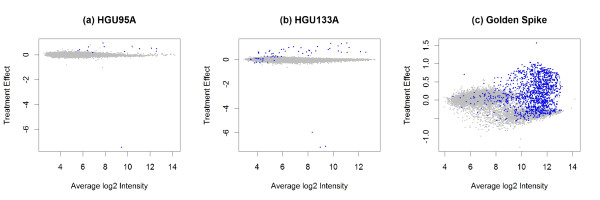
**Gaussian mixture model from PLLM**. The plot of average intensity vs. treatment effect as estimated in the PLLM approach is used to identify underlying components in a Gaussian mixture model. The blue points correspond to the known spiked-in genes in each of the three datasets. The Golden Spike dataset has three main components, with one predominantly corresponding to the spiked-in genes. In the other datasets the components are not as clear.

Lemieux (2006) proposed a probe-level linear model (PLLM) [19]. It uses a linear model to estimate the treatment effect directly, using information at the probe level. The PLLM approach begins with the simple model:(19)

for each gene *k*, where *Y*_*ijl *_is as defined following Equation 1, *α*_*l *_is a combination of the expression level of the gene across arrays and specific probe affinities, and *T*_*i *_is the effect of treatment level *i*. The treatment effect is estimated using standard least-square procedures. We plot these against the average log probeset intensities to reveal a Gaussian mixture model, as in Figure 1. The original PLLM paper [19] used the Golden Spike data, and Figure 1c shows three clusters of points for these data, one of which appears to coincide with the spike-in probesets.

To fit the Gaussian mixture, after using such plots to decide the number of components to include, we used the implementation in the R package mclust [20]. To test for differential expression, we use as the test statistic the conditional probability that the gene belongs to the most extreme cluster (in terms of the absolute treatment effect) from the Gaussian mixture model.

One drawback to this model is that it can not be automated. One has to manually choose the number of components to use in the Gaussian mixture model by looking at these plots. It is not always clear how many components to choose for the mixture model (see Figure 1ab). Based on recommendations from Lemieux (personal communication), anywhere in the order of 5-15 components seems reasonable.

#### PLW

The probe-level locally moderated weighted median-t method (PLW) uses a hierarchical Bayes model to obtain a moderated, weighted *t*-statistic for each PM probe [21,22]. With the background-corrected, quantile-normalized data, a weighted, moderated test statistic is calculated for each probe. The test statistic for whether or not gene *k *is differentially expressed is then calculated by taking the median of the moderated test statistics for each probe within gene *k*. This method has been implemented in the R package plw [21,22].

#### FC

For purposes of comparison, the naive fold change (FC) across treatments is also considered as a test statistic in identifying differentially expressed genes. For this study, the (log) fold change was calculated as the numerator of Equation 3, i.e., *c'*, where *c *and  are as previously described in the PLM discussion.

#### RMANOVA

The robustified MANOVA (RMANOVA) approach is a probe-level model based on the framework of multivariate analysis of variance [23], fit on a per-gene basis. For a given gene, let *y*_*j*(*i*) _be the vector of background-corrected quantile-normalized log-scale perfect match intensities on array *j *within treatment level *i*. For treatment level *i*, let *μ*_(*i*) _represent the corresponding vector of expected values for treatment level *i*. Similarly, let *μ *be the corresponding vector of expected values across all treatment levels. In the two-treatment case (of interest here), the null hypothesis of interest is *H*_0_: *μ*_(1) _= *μ*_(2)_.

Let the vector  be a robust estimate of *μ*_(*i*)_, obtained by taking the component-wise median across all *y*_*j*(*i*) _for treatment level *i*. Similarly, let  be a robust estimate of *μ*, obtained by taking the component-wise median across all *y*_*j*(*i*) _for all treatment levels. Let *n*_*i *_be the number of arrays in treatment level *i*. Then the within-treatment and between-treatment error matrices are estimated respectively as:(22)

and:(23)

Here the sum and median are applied component-wise.

Two test statistics are proposed by Xu and Cui (2008) [23], referred to here as RMANOVA1 and RMANOVA2, respectively:(24)

Here again the median is applied component-wise. Both of these test statistics are constructed with the same philosophy of the F-statistic in a one-way ANOVA model, as the ratio of between-treatment to within-treatment error. However, unlike the ANOVA F-statistic, the numerators of these test statistics are not directly related to the log fold change. Still, larger test statistics are taken as greater evidence of differential expression. Xu and Cui (2008) [23] provide Matlab functions for this method, but we use a custom R implementation for convenience in comparison.

#### FIRSTP

Rubin (2009) proposed a "first principles" (FIRSTP) probe-level approach to testing for differential expression [24]. For a given gene, let *Y*_*ijl *_be the background-corrected quantile-normalized log-scale perfect match intensity for probe *l *of the gene on array *j *under treatment level *i*. For each probe *l *of the gene, the FIRSTP approach is based on the simple model:(25)

where *μ*_*l *_is the population mean for probe *l*, and *T*_*i*, *l *_is the effect of treatment level *i *for probe *l*. Using traditional parametric ANOVA methods, a p-value (*p*_*l*_) is obtained for the null hypothesis *H*_0_: *T*_1, *l *_= *T*_2, *l*_, for each probe *l *of the gene. (We encountered undefined *p*_*l *_values for the occasional probes where the *Y *values were the same on all arrays. This was more common with very small numbers of arrays. Our strategy was to reset these *p*_*l *_values to 1 to represent no evidence of differential expression of the probe.)

For the overall test of differential expression for the gene, the test statistic for the gene is the median (over all probes *l*) of 1 *- p*_*l*_. Larger values of the test statistic are indicative of greater evidence of differential expression. This FIRSTP approach is similar in spirit to the PLW method (because both utilize medians of probe-level test statistics), but differs in that the "first principles" philosophy limits the number of assumptions and model components. We used a custom R implementation of this FIRSTP method.

#### PUMA

Propagating Uncertainty in Microarray Analysis, or PUMA, accounts for uncertainty of the measured value of each gene when doing these experiments, and includes a probe-specific parameter in a probabilistic model [25-27]. The model accounts for information from both the perfect match (PM) and mismatch (MM) probes, instead of treating MM probes as background.

The first step of PUMA is to preprocess the raw microarray data using a method called multi-chip modified gamma Model for Oligonucleotide Signal, or multi-mgMOS. This method uses a hierarchical Bayes model to obtain the expression level for each gene.

After this preprocessing, PUMA uses a hierarchical model to obtain the posterior distribution on *μ*_*i*_, the mean expression value of a gene for treatment level *i*. Since this posterior distribution cannot be written in closed form, an EM algorithm (using MCMC) is used to approximate the distribution. The test statistic for each gene is then the probability that the gene is up-regulated (or down-regulated); i.e., the probability that *μ*_1 _>*μ*_2 _(or *μ*_2 _>*μ*_1_), is then calculated. These test statistics can be converted into a "p-like-value," which is the probability that each gene is differentially expressed. The p-like-values are more comparable to the p-values returned from other methods, in that smaller values represent stronger evidence of differential expression. These methods have been implemented in the R package puma [28].

### Testing

In order to understand how well each of the previously defined statistical models can identify differentially expressed genes, each was compared simultaneously by considering the true positive rate (TPR) and the false positive rate (FPR) in the three spike-in datasets described previously. For each model, a test statistic was obtained for each gene and the absolute values of these test statistics were sorted in increasing order. The TPR and FPR values were defined as in Bolstad (2004) [17]: For the entire set of sorted test statistics, count the number of spike-in genes that are called significant (i.e. that are in the set being considered) and divide that by the total number of spike-in genes. Call that the TPR for the first set of sorted test statistics. Count the number of non-spike-in genes called significant and divide that by the total number of genes that are not spiked-in. Call this the FPR for the first set of sorted test statistics. Then omit the test statistic with the smallest absolute value and obtain a TPR and FPR for that subset of test statistics. Repeat this process by removing the test statistic with the smallest absolute value each time, obtaining TPRs and FPRs for each subset of test statistics. For the last iteration, the subset will only contain one test statistic. The total number of TPR and FPR values obtained will be equal to the total number of genes in the dataset.

For each spike-in dataset, a receiver operating characteristic (ROC) curve was created to visually assess how well each model performed. The ROC curve is a plot of TPR vs. FPR. A higher curve indicates higher performance in identifying differentially expressed genes. "Higher" here can be assessed both graphically (with the ROC curve) and numerically (with the AUC, or area under the ROC curve).

Because our focus is performance in small-sample studies, we also looked at subsets of the spike-in studies. For example, the HGU95A dataset has four arrays in each of two treatments. In addition to this 4 × 4 comparison, we also tested for differential expression in all 16 3 × 3 and all 36 2 × 2 comparisons. For each method fit, the TPR and FPR were averaged across all 16 3 × 3 comparisons, and separately averaged across all 36 2 × 2 comparisons. The HGU133A and Golden Spike datasets both have three arrays in each of two treatments. In addition to their 3 × 3 comparisons, we averaged the TPR and FPR across their respective 9 2 × 2 comparisons.

#### Results

**Figure 2:**
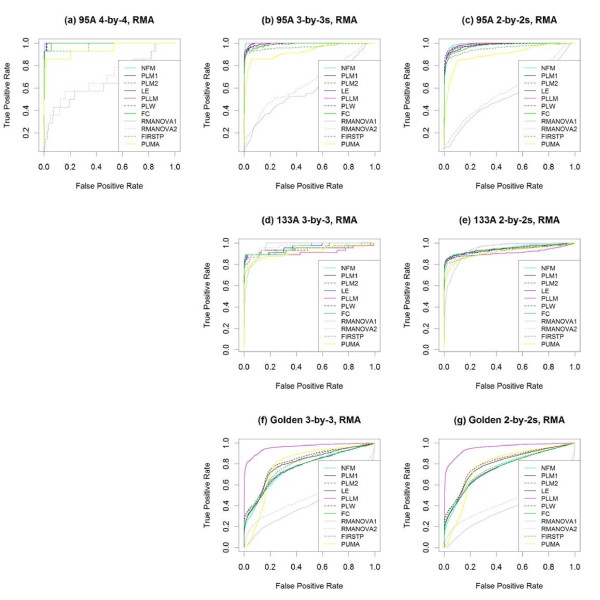
**ROC curves for RMA-preprocessed data**. With RMA preprocessing, the performance of several methods testing for differential expression is compared using **(a) **the full 4 × 4 comparison of the HGU95A spike-in data, as well as the averages across **(b) **all 3 × 3 and **(c) **all 2 × 2 subsets. The methods are also compared using **(d) **the full 3 × 3 and **(e) **the average of all 2 × 2 comparisons of the HGU133A spike-in data, as well as using **(f) **the full 3 × 3 and **(g) **the average of all 2 × 2 comparisons of the Golden Spike spike-in data.

**Figure 3:**
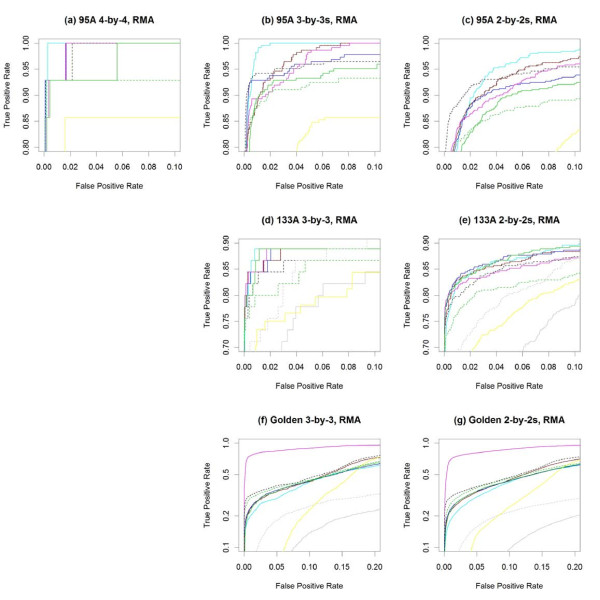
**Partial ROC curves for RMA-preprocessed data**. **(a-h) **Partial ROC curves from Figure 2 to focus on portions of greatest interest - low false positive and high true positive rates. Note that the vertical axes in **(f) **and **(g) **are on the log scale to facilitate visualization. The same color legend of Figure 2 applies here.

Figure 2 and Table 1 summarize the performance of the various methods on all three spike-in data sets plus the average performance across their subsets, using RMA preprocessing [11] for all methods, except for PUMA, which relies on multi-mgMOS preprocessing [25-27]. Figure 3 "zooms in" on the same ROC curves to focus on performance at lower false positive rates (FPR). For methods with higher ROC curves at low FPR, the genes at the top of their ranked lists (by test statistic) are more likely to be the truly differentially expressed genes than are those at the top of the other methods' ranked lists.

**Table 1:** Area Under Curve (AUC) values for each model on each data subset, using RMA preprocessing.

	HGU95A			HGU133A		Golden Spike	
	4 × 4	3 × 3s	2 × 2s	3 × 3	2 × 2s	3 × 3	2 × 2s
NFM	0.9993	0.9987	0.9922	0.9510	0.9488	0.7611	0.7568
PLM1	0.9982	0.9961	0.9896	0.9447	0.9471	0.8009	0.7977
PLM2	0.9982	0.9961	0.9895	0.9447	0.9470	0.8009	0.7977
LE	0.9985	0.9941	0.9817	0.9597	0.9397	0.7658	0.7600
PL-LM	0.9982	0.9952	0.9864	0.9168	0.9110	0.9619	0.9553
PLW	0.9982	0.9906	0.9854	0.9466	0.9375	0.8208	0.8138
FC	0.9953	0.9891	0.9764	0.9571	0.9403	0.7679	0.7622
RMANOVA1	0.6638	0.5854	0.5320	0.9665	0.9413	0.4142	0.3816
RMANOVA2	0.6935	0.6492	0.5885	0.9713	0.9580	0.4922	0.4577
FIRSTP	0.9753	0.9660	0.9553	0.9409	0.9244	0.7983	0.7746
PUMA	0.9435	0.9276	0.9019	0.9133	0.9143	0.7901	0.7769

**Figure 4:**
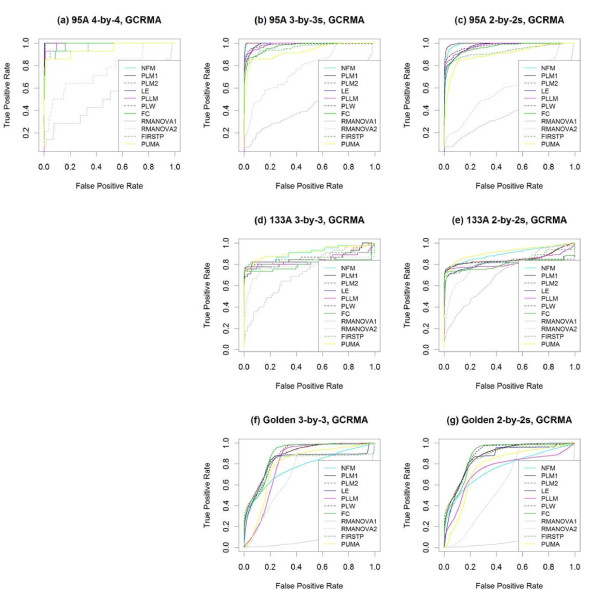
**ROC curves for GCRMA-preprocessed data**. With GCRMA preprocessing, the performance of several methods testing for differential expression is compared using **(a) **the full 4 × 4 comparison of the HGU95A spike-in data, as well as the averages across **(b) **all 3 × 3 and **(c) **all 2 × 2 subsets. The methods are also compared using **(d) **the full 3 × 3 and **(e) **the average of all 2 × 2 comparisons of the HGU133A spike-in data, as well as using **(f) **the full 3 × 3 and **(g) **the average of all 2 × 2 comparisons of the Golden Spike spike-in data.

**Figure 5:**
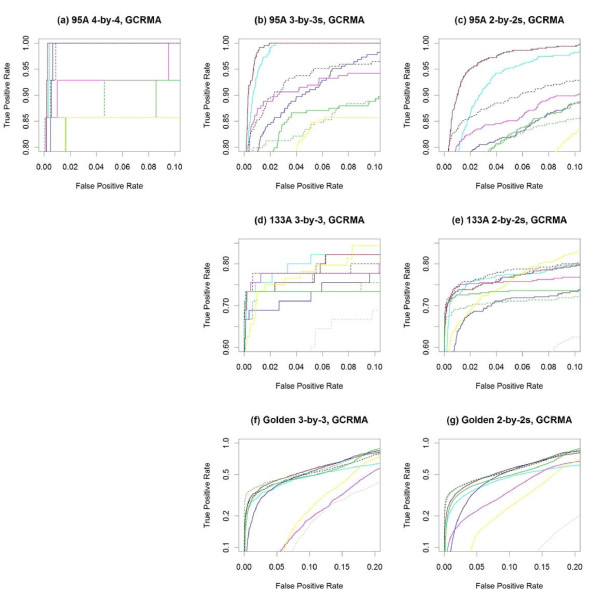
**Partial ROC curves for GCRMA-preprocessed data**. **(a-h) **Partial ROC curves from Figure 4 to focus on portions of greatest interest - low false positive and high true positive rates. Note that the vertical axes in **(f) **and **(g) **are on the log scale to facilitate visualization. The same color legend of Figure 4 applies here.

Figure 4 and Table 2 summarize the performance of the various methods on all three spike-in data sets plus the average performance across their subsets, using GCRMA preprocessing [12] for all methods, except for PUMA, which relies on multi-mgMOS preprocessing [25-27]. Figure 5 "zooms in" on the same ROC curves to focus on performance at lower false positive rates (FPR).

**Table 2:** Area Under Curve (AUC) values for each model on each data subset, using GCRMA preprocessing.

	HGU95A			HGU133A		Golden Spike	
	4 × 4	3 × 3s	2 × 2s	3 × 3	2 × 2s	3 × 3	2 × 2s
NFM	0.9991	0.9979	0.9907	0.9080	0.8989	0.7705	0.7618
PLM1	0.9994	0.9988	0.9960	0.8604	0.8531	0.8809	0.8806
PLM2	0.9994	0.9988	0.9959	0.8607	0.8549	0.8794	0.8783
LE	0.9985	0.9892	0.9702	0.8165	0.8076	0.8213	0.8582
PL-LM	0.9920	0.9873	0.9744	0.8356	0.8227	0.8075	0.7480
PLW	0.9988	0.9859	0.9762	0.8473	0.8294	0.8795	0.8918
FC	0.9801	0.9713	0.9616	0.8006	0.8004	0.8871	0.8868
RMANOVA1	0.4838	0.4356	0.3864	0.7273	0.7066	0.1616	0.1319
RMANOVA2	0.7556	0.7712	0.6028	0.7867	0.7849	0.7468	0.6299
FIRSTP	0.9725	0.9611	0.9282	0.8500	0.8347	0.8135	0.8680
PUMA	0.9435	0.9276	0.9019	0.9133	0.9143	0.7901	0.7769

The NFM method is consistently among the top performers at low FPR, except in the Golden Spike data. (The poorer performance in the Golden Spike data is examined in the two following paragraphs.) The PLM methods perform well in the HGU95A data, and are among the best at low FPR for the Golden Spike data with GCRMA preprocessing. The LE method has a slightly variable performance, doing well in the HGU95A data, and among the best at low FPR for the HGU133A data with RMA preprocessing, but among the worst at low FPR for the HGU133A data with GCRMA preprocessing. The PLLM method is more variable, as it is clearly the best performer on the Golden Spike data with RMA preprocessing, and among the best at low FPR on the HGU133A data with GCRMA preprocessing, but among the worst at low FPR for the Golden Spike data with GCRMA preprocessing. The PLW method is among the best for the Golden Spike data with GCRMA preprocessing, and at low FPR for the HGU133A data with GCRMA preprocessing. The FC method is among the best for the Golden Spike data with GCRMA preprocessing and at low FPR for the HGU133A data with RMA preprocessing, but among the worst at low FPR for subsets of the HGU95A and HGU133A data with GCRMA preprocessing. RMANOVA tends to be among the lowest performers, but gives the best overall performance on the HGU133A data with RMA preprocessing (and even there, it is a low performer at low FPR). The FIRSTP method gives a variable performance, and is never the best or worst overall. PUMA is the top overall performer on the HGU133A data with GCRMA preprocessing, but there (and consistently in the other data sets) it is among the low performers at low FPR.

**Figure 6:**
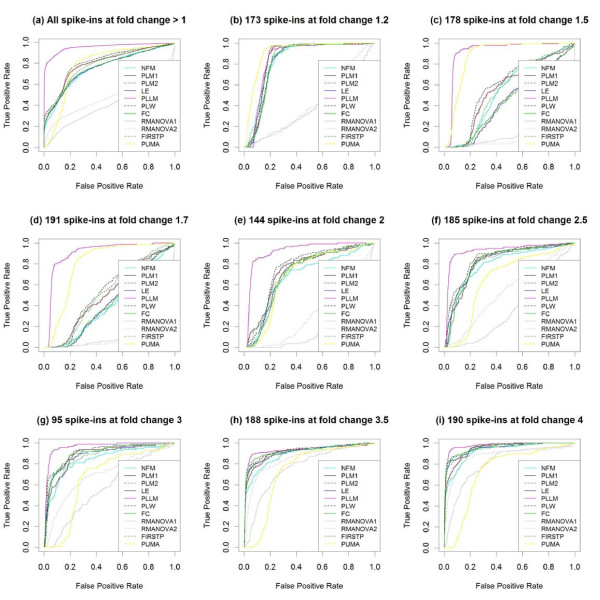
**ROC curves for Golden Spike data, by known fold chang**. With RMA preprocessing, the performance of several methods testing for differential expression is compared using the full 3 × 3 comparison of the Golden Spike spike-in data, treating the eight levels of reported spiked-in fold changes separately. Several methods have difficulty detecting differential expression for spiked-in genes with fold changes 1.5 and 1.7 in particular.

**Figure 7:**
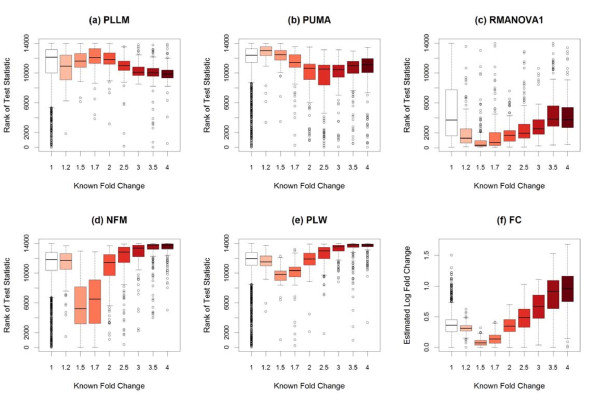
**Rank of test statistics in Golden Spike data, by fold change**. **(a-e) **With RMA preprocessing in the full 3 × 3 comparison of the Golden Spike spike-in data, the overall ranks of test statistics from five methods are compared to the spiked-in fold changes. (The known fold change of 1 corresponds to non-spiked-in genes.) Both PLLM and PUMA show an overall drop in ranks for higher fold changes, while RMANOVA, NFM, and PLW show an overall increase in ranks for higher fold changes. There is a clear overall drop in NFM and PLW ranks for spiked-in genes with fold changes 1.5 and 1.7. **(f) **With RMA preprocessing in the same Golden Spike comparison, the distributions of estimated log fold changes are compared to the known spiked-in fold changes. There is a clear drop in estimated log fold changes for spiked-in genes with known fold changes 1.5 and 1.7. This contributes to the poorer performance of the fold-change-based methods at these fold change levels.

Because the Golden Spike data have large numbers of genes spiked in at each of several known fold changes, the performance of these statistical methods can be compared in identifying the spiked-in genes by fold change. Figure 6 summarizes the performance of the various methods on the full 3 × 3 Golden Spike comparison with RMA preprocessing, for identifying genes spiked-in at different fold-changes. There appear to be four levels of performance on the Golden Spike data - PLLM is generally superior for all fold-changes except 1.2 (and this superiority may be attributable to the unique pattern observed in Figure 1c), PUMA performs worse for larger fold-changes, RMANOVA performs poorly in general (with improvement for larger fold-changes), and the other methods tend to perform roughly similarly (with noticeably poorer performance at fold-changes 1.5 and 1.7). Figure 7 considers these four levels of performance, with NFM and PLW as examples of the final level. The distributions of overall ranks of test statistics within each method are compared based on the spiked-in fold-changes. While PLLM and PUMA show an overall drop in ranks for higher fold changes, the expected overall increase in ranks for higher fold changes is observed for RMANOVA, NFM, and PLW.

The methods comprising this fourth level of performance (NFM, PLM, LE, PLW, FC, and FIRSTP) all share a common characteristic, that their test statistics are related to the simple estimated log fold change. This is due to their being based on traditional ANOVA-type models. (The PLLM method begins with the ANOVA-type model in Equation 10, but the test statistic is actually based on the result of the subsequent Gaussian mixture model.) When this estimated log fold change is low, the test statistics for these methods tend to be lower. This is what caused these methods' performance to drop in the Golden Spike data at fold-changes 1.5 and 1.7. Figure 7f shows that at these fold-changes, the estimated log fold changes were noticeably and systematically lower.

## Implementation

### All Methods - Bovine NT Application

**Figure 8:**
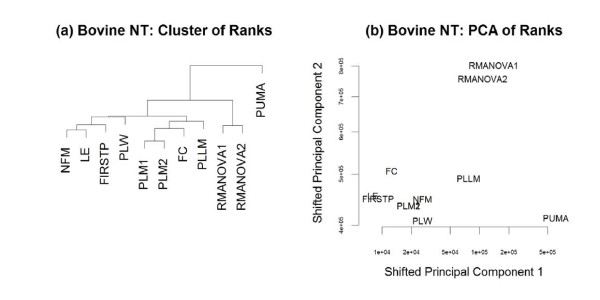
**Comparison of results on Bovine NT data**. **(a) **The relationships among the methods considered here are visualized by clustering the vectors of test statistic ranks within each method when applied to the bovine NT data. **(b) **A biplot (based on the first two principal components of ranks of test statistics within method) visualizes the same relationships. The principal components were shifted for visualization purposes, to allow both axes to be on the log scale.

All probe-level and probeset-level methods discussed previously were applied to the motivating bovine NT data, with RMA preprocessing for the non-PUMA methods. The top 500 genes (based on ranked test statistics) from each method were identified, with a union of 2,432 genes across the methods. The overall ranks of these 2,432 genes within each method were used to compare the results of each method while allowing for scale differences between test statistics from each method. Figure 8a visualizes the agglomerative nesting clustering of the methods (based on overall ranks of these 2,432 genes), using Manhattan distance with average linkage. The biplot in Figure 8b visualizes the first two principal components for these ranks of test statistics within method. This principal components analysis made use of the princomp2 function in the msProcess R package [29], allowing for "wide" data such as this, where the number of genes far exceeds the number of methods. Figure 8 supports what was seen in Figures 6 and 7, that in terms of ranking genes, the PUMA, RMANOVA, and PLLM approaches each stand apart as performing quite differently from the other fold-change-based methods.

### NFM P-Value Calculation

Based on its consistently strong performance on the spike-in data, including small-sample subsets, NFM was applied to the motivating bovine NT data. To assess statistical significance in this application, p-values (with subsequent multiple testing adjustment) are necessary. The F-statistics from NFM have a theoretical  sampling distribution, where *g *is the number of treatment levels and *n*_*i *_is the number of samples within treatment level *i*. In other words, when the null hypothesis *H*_0_: *T*_1 _= *T*_2 _is true, the F-statistics will theoretically have this approximate distribution, and p-values could be obtained as tail probabilities. The validity of this distributional assumption can be assessed using the F-statistics for the non-spiked-in genes in the three spike-in datasets, because the null hypothesis is known to be true for these genes.

**Figure 9:**
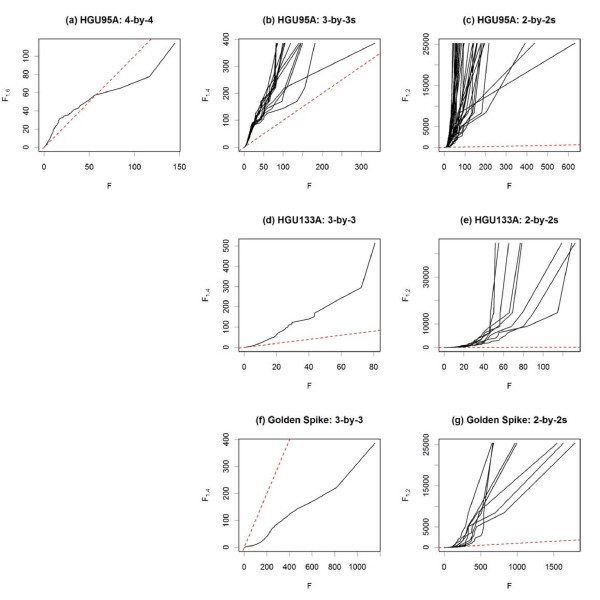
**F-quantile plots of the NFM test statistics of non-spike-in genes**. Quantile plots for the HGU95A, HGU133A, and Golden Spike datasets show that the theoretical F-distribution (vertical axis) is not a good approximation for the sampling distribution of the observed NFM test statistics (horizontal axis). A solid black curve represents the quantile plot for the test statistics of the non-spike-in genes in each full data and subset comparison. Deviations from the dashed red reference line of equality indicate departures from the theoretical distribution.

The F-quantile plots in Figure 9 summarize this distributional assumption assessment. The 4 × 4 HGU95A comparison (Figure 9a) is the only case where the theoretical distribution is reasonably close to the observed distribution. The 3 × 3 Golden Spike comparison (Figure 9f) is the only case where the observed distribution is longer-tailed than the theoretical distribution. In all other cases, the distributions of the observed F-statistics are grossly short-tailed compared to the theoretical sampling distributions. This will affect p-value calculations, as the tail probabilities (p-values) from these theoretical sampling distributions will not be small enough to allow claims of statistical significance, even for these spike-in datasets.

An alternative sampling distribution is generated by a permutation approach. Existing permutation or bootstrap approaches permute or resample treatment labels, and the p-value for each gene is the proportion of all resamples producing a test statistic for the gene at least as extreme as the original test statistic for the gene. An implementation of such an approach [30] is found in the R package multtest [31]. Using this existing approach, the smallest p-value possible will be the inverse of the number of resamples. For this reason, such a per-gene permutation-based approach with very small sample sizes (as in the current application) will not yield small enough p-values to claim statistical significance, because the number of possible permutations is low.

For example, in the HGU133A dataset, there are 3 "control" and 3 "treatment" arrays under consideration, so there are 6!*/*(3!·3!) = 20 possible permutations of treatment labels. Due to the one-sided nature of the NFM test statistic, there are actually only half that number of permutations that will give unique (or non-redundant) test statistics for any given gene. For example, the treatment labels (C,C,C,T,T,T) and (T,T,T,C,C,C) will produce identical F-statistics for a given gene, where C represents control and T represents treatment. Then the smallest p-value using this per-gene permutation approach is 1/10, and no claims of significance can be made, particularly after adjusting for multiple hypothesis testing.

Instead, we propose to use the same sampling distribution for the test statistics of all genes in an experiment. If the  distribution had been a good approximation for the sampling distribution of the NFM test statistics for which the null hypothesis was known to be true, then that same theoretical distribution would have been used for the p-value calculation of all genes in the experiment. Since this theoretical distribution was shown to be a poor approximation (Figure 9), we generate an alternative, more appropriate distribution using permutations.

For a given experiment, we enumerate all treatment label permutations, and for each permutation we calculate the NFM test statistic for each gene. Our sampling distribution is the collection of test statistics for all genes across all permutations of treatment labels. Then for each gene, the NFM p-value is the proportion of all test statistics in this collection that are at least as extreme (large) as the gene's test statistic using the original treatment labels. The false discovery rate [32] is controlled by converting these raw p-values to q-values using the R package qvalue [33].

**Figure 10:**
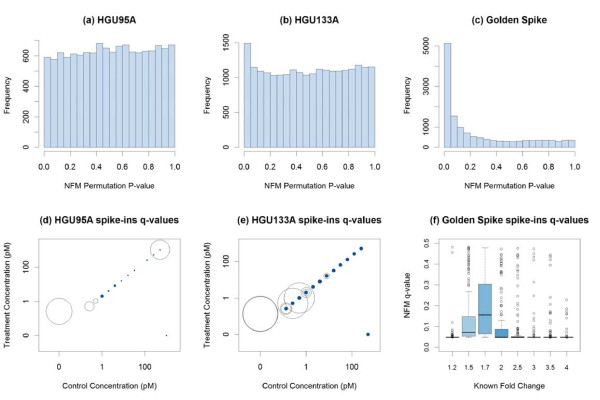
**Significance plots of NFM permutation results**. **(a-c) **Histograms of NFM permutation p-values for the full data comparisons in each of the three spike-in datasets. **(d-e) **Bubble plots for the spike-in genes of the HGU95A and HGU133A spike-in datasets. The horizontal and vertical axes are the spike-in concentrations for the control and treatment conditions, with axis tick marks on the log scale. The size of the plotting character for each spike-in gene is proportional to the corresponding q-value (converted from NFM permutation p-values). Q-values less than 0.1 are represented as closed blue dots, while q-values greater than 0.1 are represented as open circles. Statistical significance (q-value < 0.1) is more common for genes with higher control and treatment concentrations. **(f) **The distribution of calculated NFM q-values (converted from NFM permutation p-values) by spiked-in fold-change for the spike-in genes of the Golden Spike dataset.

Figure 10 summarizes the significance results for the three spike-in datasets, using the full comparison (4 × 4 or 3 × 3) for each. As the number of spike-in genes increases (14 in HGU95A, 45 in HGU133A, 1,331 in Golden Spike), the distribution of NFM permutation p-values becomes less uniform (Figure 10a-c). For the HGU95A and HGU133A datasets, statistical significance (q-value *<*0.1) is more common for spike-in genes with higher control and treatment concentrations (Figure 10d-e). These concentrations are as reported by the dataset publisher [6]. The only notable exception is the 407_at probeset in the HGU95A dataset, represented by a large open circle in the upper-right of Figure 10d. For this probeset, the control concentration is 512 pM, the treatment concentration is 1024 pM, and the NFM q-value is 0.43. The dataset publisher reports that certain probe pairs for this 407_at probeset have been found to perform poorly [6], which may explain this anomalous result. For the Golden Spike dataset, the spike-in genes' q-values tend to be small (Figure 10f), except for the aforementioned known fold-change values of 1.5 and 1.7, where the estimated log fold-changes were quite small (Figure 7f).

#### NFM Convergence

The iterative REML process used by the NFM method to obtain test statistics can converge slowly, and for some genes it does not by default converge in R. However, model convergence for these same genes in SAS [34] is not problematic. We have found that the non-convergence in R can be eliminated by trivial rounding of the log-scale intensity data (to the fifth decimal place, for example).

#### NFM Bovine NT Application

In applying the NFM method to the motivating bovine NT data, we used a non-specific filter [35,36], and only calculated q-values for the 16,706 genes with expression above 20 on at least two of the seven arrays. With RMA preprocessing, this resulted in 584 significant genes (FDR .05). As a check for biological relevance, we then tested for over-representation of biological process Gene Ontology terms [37] using a conditional hypergeometric test [38,39].

The clone samples in these motivating bovine NT data were the result of somatic cell nuclear transfer (SCNT). Despite improvements in recent years, bovine somatic cell nuclear transfer efficiency remains quite low. While pregnancy rates of transferred SCNT embryos approach those of transferred in vitro fertilized (IVF) embryos, pregnancy loss throughout gestation, and particularly in the first trimester, is much higher for SCNT pregnancies. The placental structure in SCNT pregnancies is often remarkably different in bovine SCNT pregnancies, with significantly fewer and significantly larger cotyledons and caruncles [40]. In addition, SCNT fetuses that are lost mid-gestation as well as a portion of those that reach term demonstrate developmental abnormalities and metabolic problems [41-43].

An evaluation of the most significantly over-represented biological processes (from the conditional hypergeometric test) provides interesting insight regarding aberrations in SCNT cotyledons. Three main categories of over-represented biological processes were identified - cell cycle regulation, metabolism, and early developmental processes. Cell cycle arrest was the most significantly over-represented biological process. The most significantly over-represented metabolism ontologies were RNA processing and sphingolipid metabolic process. Among the most significantly over-represented developmental processes were brain development, neuron maturation, hindlimb morphogenesis, in utero embryonic development, and muscle organ development. These all fit very well with the abnormal phenotypes we see in SCNT pregnancies.

## Conclusion

Of the statistical methods considered here for small-sample gene expression data, none was shown to be consistently superior, but a few interesting conclusions can be made. The fold-change-based methods (NFM, PLM, LE, PLW, FC, and FIRSTP) tended to be more consistent in their performance and provided the expected increase in test statistic ranks for higher fold-changes. By contrast, the non-fold-change-based methods (PLLM, RMANOVA, and PUMA) tended to be more variable in performance, and two of them (PLLM and PUMA) showed an unexpected decrease in test statistic ranks for higher fold-changes.

For small-sample gene expression experiments, the nested factorial model (NFM) was shown to be a competitive statistical approach for identifying differentially expressed genes, particularly at low false positive rates. The NFM method uses a permutation approach to calculate a p-value for each gene. These permutations add computational expense, but this expense must be taken in perspective. Small-sample studies (for which NFM is of immediate interest) are common in applications where physical samples are either scarce or prohibitively expensive. When a research team is unable to acquire additional biological samples or chips to perform a larger-sample gene expression study, they will have to compensate with additional waiting time. For the HGU95A spike-in dataset, with 12,626 genes on 4 control and 4 treatment arrays, the total runtime to acquire the permutation p-values was about 38 hours on a typical desktop PC. The HGU133A spike-in dataset, with 22,300 genes on 3 control and 3 treatment arrays, required a runtime of about 16 hours. The Golden Spike spike-in dataset, with 14,010 genes on 3 control and 3 treatment arrays, required a runtime of about 10 hours. The runtime for the bovine NT dataset, with 24,128 genes on 3 control and 4 treatment arrays, was about 50 hours. When a research team faces insufficient resources for a larger-sample gene expression study, these runtimes should not seem particularly onerous.

The NFM methodology described here, including the p-value permutation approach and trivial rounding in cases of initial non-convergence, has been implemented in R code (affyNFM) freely available from the first author's website at http://www.stat.usu.edu/~jrstevens. A tutorial document (including a reproducible example) is provided on the same website.

We conclude with a caveat for those who may attempt too much analysis with too little data. The development and availability of statistical methodology specifically for small-sample gene expression studies (such as the NFM here) should not be taken as encouragement of poor design. Our motivating example involved data where it was extremely expensive to obtain a single biological sample (of the clone). In practice, we would have preferred a larger sample size to provide more information on population-level biological variability. It was not merely expensive to acquire the chips, and not merely inconvenient to acquire additional biological replicates. We emphasize that sufficient sample size for useful inference should remain a guiding principle of experimental design in gene expression studies.

## Authors' contributions

JRS conceived of the study, supervised the M.S. thesis of JLB, prepared the affyNFM implementation, and drafted the manuscript for publication. JLB developed the R code to execute and compare the various statistical methods. KIA and KLW designed, executed, and provided interpretation and context for the original bovine NT study which motivated this work. All authors read and approved the final manuscript.

## References

[B1] Lockhart DJ Dong H Byrne MC Follettie MT Gallo MV Chee MS Mittmann M Wang C Kobayashi M Horton H Brown EL Expression monitoring by hybridization to high-density oligonucleotide arrays Nature Biotechnology 1996 14 10.1038/nbt1296-1675 9634850

[B2] Aston KI Li GP Hicks BA Sessions BR Pate BJ Hammon DS Bunch TD White KL The developmental competence of bovine nuclear transfer embryos derived from cow versus heifer cytoplasts Animal Reproduction Science 2006 95 234 243 10.1016/j.anireprosci.2005.10.011 16324805

[B3] Aston KI Li GP Hicks BA Sessions BR Pate BJ Hammon DS Bunch TD White KL Effect of the time interval between fusion and activation on nuclear state and development in vitro and in vivo of bovine somatic cell nuclear transfer embryos Reproduction 2006 131 45 51 10.1530/rep.1.00714 16388008

[B4] Aston KI Li GP Sessions BR Davis AP Winger QA Rickords LF Stevens JR White KL Global Gene Expression Analysis of Bovine Somatic Cell Nuclear Transfer Blastocysts and Cotyledons Molecular Reproduction and Development 2009 76 471 482 10.1002/mrd.20962 19062181

[B5] Gentleman R Huber W Carey VJ Irizarry RA Dudoit S Bioinformatics and Computational Biology Solutions Using R and Bioconductor 2005 New York, Springer

[B6] Affymetrix Latin Square Data for Expression Algorithm Assessment http://www.affymetrix.com/support/technical/sample_data/datasets.affx

[B7] Chen Z McGee M Liu Q Scheuermann RH A distribution free summarization method for Affymetrix GeneChip arrays Bioinformatics 2007 23 3 321 327 10.1093/bioinformatics/btl609 17148508

[B8] Choe SE Boutros M Michelson AM Church GM Halfon MS Preferred analysis methods for Affymetrix GeneChips revealed by a wholly defined control dataset Genome Biology 2005 6 R16 15693945 10.1186/gb-2005-6-2-r16 PMC551536

[B9] Irizarry RA Cope LM Wu Z Feature-level expoloration of a published Affymetrix GeneChip control dataset Genome Biology 2006 7 404 16953902 10.1186/gb-2006-7-8-404 PMC1779590

[B10] Pearson RD A comprehensive re-analysis of the Golden Spike data: Towards a benchmark for differential expression methods BMC Bioinformatics 2008 9 164 18366762 10.1186/1471-2105-9-164 PMC2324099

[B11] Irizarry RA Bolstad BM Collin F Cope LM Hobbs B Speed TP Summaries of Affymetrix GeneChip probe level data Nucleic Acids Research 2003 31 4 e15 12582260 10.1093/nar/gng015 PMC150247

[B12] Wu ZJ Irizarry RA Gentleman R Martinez Murillo F Spencer F A model-based background adjustment for oligonucleotide expression arrays Journal of the American Statistical Association 2004 99 468 909 917 10.1198/016214504000000683

[B13] R Development Core Team R: A Language and Environment for Statistical Computing 2009 R Foundation for Statistical Computing, Vienna, Austria http://www.R-project.org

[B14] Gentleman RC Carey VJ Bates DM Bolstad B Dettling M Dudoit S Ellis B Gautier L Ge Y Gentry J Hornik K Hothorn T Huber W Iacus S Irizarry R Leisch F Li C Maechler M Rossini AJ Sawitzki G Smith C Smyth G Tierney L Yang JYH Zhang J Bioconductor: Open software development for computational biology and bioinformatics Genome Biology 2004 5 R80 10.1186/gb-2004-5-10-r80 15461798 PMC545600

[B15] Pinheiro JC Bates DM Unconstrained Parameterizations for Variance-Covariance Matrices Statistics and Computing 1996 6 289 296 10.1007/BF00140873

[B16] Pinheiro JC Bates DM Mixed-Effects Models in S and S-PLUS 2000 New York, Springer

[B17] Bolstad B Probe-level model based test statistics for detecting differential expression PhD thesis 2004 University of California, Berkeley

[B18] Smyth GK Linear models and empirical Bayes Methods for assessing differential expression in microarray experiments Statistical Applications in Genetics and Molecular Biology 2004 3 1 3 10.2202/1544-6115.1027 16646809

[B19] Lemieux S Probe-level linear model fitting and mixture modeling results in high accuracy detection of differential gene expression BMC Bioinformatics 2006 7 391 16934150 10.1186/1471-2105-7-391 PMC1579233

[B20] Fraley C Raftery AE Model-based clustering, discriminant analysis, and density estimation Journal of the American Statistical Association 2002 97 611 631 10.1198/016214502760047131

[B21] Astrand M Mostad P Rudemo M Improved Covariance Matrix Estimators for Weighted Analysis of Microarray Data Journal of Computational Biology 2007 14 10 1353 1367 10.1089/cmb.2007.0078 18052774

[B22] Astrand M Mostad P Rudemo M Empirical Bayes models for multiple probe type microarrays at the probe level BMC Bioinformatics 2008 9 156 18366694 10.1186/1471-2105-9-156 PMC2358895

[B23] Xu J Cui X Robustified MANOVA with applications in detecting differentially expressed genes from oligonucleotide arrays Bioinformatics 2008 24 8 1056 1062 10.1093/bioinformatics/btn053 18316342

[B24] Rubin RA A first principles approach to differential expression in microarray data analysis BMC Bioinformatics 2009 10 292 19758448 10.1186/1471-2105-10-292 PMC2749840

[B25] Liu X Milo M Lawrence ND Rattray M A tractable probabilistic model for Affymetrix probe-level analysis across multiple chips Bioinformatics 2005 21 18 3637 3644 10.1093/bioinformatics/bti583 16020470

[B26] Liu X Milo M Lawrence ND Rattray M Probe-level measurement error improves accuracy in detecting differential gene expression Bioinformatics 2006 22 17 2107 2113 10.1093/bioinformatics/btl361 16820429

[B27] Liu X Lin KK Andersen B Rattray M Including probe-level uncertainty in model-based gene expression clustering BMC Bioinformatics 2007 8 98 10.1186/1471-2105-8-98 PMC1847531 17376221

[B28] Pearson RD Liu X Sanguinetti G Milo M Lawrence ND Rattray M puma: a Bioconductor package for Propagating Uncertainty in Microarray Analysis BMC Bioinformatics 2009 10 211 10.1186/1471-2105-10-211 19589155 PMC2714555

[B29] Gong L Constantine W Chen YA An S-PLUS module for protein mass spectra processing and classification 2008 TIBCO Software Inc http://www.insightful.com/services/research/proteome/default.asp

[B30] Westfall PH Young SS Resampling-based multiple testing: Examples and methods for p-value adjustment 1993 New York, John Wiley and Sons

[B31] Pollard KS Dudoit S Laan MJ van der Gentleman R, Carey VJ, Huber W, Irizarry RA, Dudoit S Multiple Testing Procedures: the multtest Package and Applications to Genomics Bioinformatics and Computational Biology Solutions Using R and Bioconductor 2005 Springer 249 271 full_text

[B32] Benjamini Y Hochberg Y Controlling the false discovery rate: a practical and powerful approach to multiple testing Journal of the Royal Statistical Society, Series B 1995 57 289 300

[B33] Storey J Tibshirani RJ Statistical significance for genomewide studies Proceedings of the National Academy of Sciences 2003 57 289 300 10.1073/pnas.1530509100 PMC170937 12883005

[B34] Version 9.1 of the SAS System for Windows Copyright 2009 SAS Institute Inc

[B35] Scholtens D von Heydebreck A Gentleman R, Carey VJ, Huber W, Irizarry RA, Dudoit S Analysis of Differential Gene Expression Studies Bioinformatics and Computational Biology Solutions Using R and Bioconductor 2005 Springer 229 248 full_text

[B36] Hackstadt AJ Hess AM Filtering for Increased Power for Microarray Data Analysis BMC Bioinformatics 2009 10 11 19133141 10.1186/1471-2105-10-11 PMC2661050

[B37] The Gene Ontology Consortium Gene Ontology: tool for the unification of biology Nature Genetics 2000 25 25 29 10.1038/75556 10802651 PMC3037419

[B38] Gentleman R Scholtens D Ding B Carey VJ Huber W Gentleman R, Carey VJ, Huber W, Irizarry RA, Dudoit S Case Studies Using Graphs on Biological Data Bioinformatics and Computational Biology Solutions Using R and Bioconductor 2005 Springer 369 394 full_text

[B39] Alexa A Rahnenfuhrer J Lengauer T Improved scoring of functional groups from gene expression data by decorrelating GO graph structure Bioinformatics 2006 22 13 1600 1607 10.1093/bioinformatics/btl140 16606683

[B40] Edwards JL Schrick FN McCracken MD van Amstel SR Hopkins FM Welborn MG Davies CJ Cloning adult farm animals: a review of the possibilities and problems associated with somatic cell nuclear transfer American Journal of Reproductive Immunology 2003 50 113 123 10.1034/j.1600-0897.2003.00064.x 12846674

[B41] Heyman Y Chavatte-Palmer P LeBourhis D Camous S Vignon X Renard JP Frequency and occurrence of late-gestation losses from cattle cloned embryos Biology of Reproduction 2002 66 6 13 10.1095/biolreprod66.1.6 11751257

[B42] Hill JR Roussel AJ Cibelli JB Edwards JF Hooper NL Miller MW Thompson JA Looney CR Westhusin ME Robl JM Stice SL Clinical and pathological features of cloned transgenic calves and fetuses (13 case studies) Theriogenology 1999 51 1451 1465 10.1016/S0093-691X(99)00089-8 10729073

[B43] Hill JR Burghardt RC Jones K Long CR Looney CR Shin T Spencer TE Thompson JA Winger QA Westhusin ME Evidence for placental abnormality as the major cause of mortality in first-trimester somatic cell cloned bovine fetuses Biology of Reproduction 2000 63 1787 1794 10.1095/biolreprod63.6.1787 11090450

